# Reduced cortisol metabolism drives hypercortisolism in critical illness

**DOI:** 10.1186/cc10762

**Published:** 2012-03-20

**Authors:** E Boonen, H Vervenne, P Meersseman, L Mortier, YM Vanwijngaerden, I Spriet, L Langouche, I Vanhorebeek, G Van den Berghe

**Affiliations:** 1 KU Leuven, Belgium; 2 University Hospitals, Leuven, Belgium; 3 Virga Jesse Hospital, Hasselt, Belgium

## Introduction

Critical illness is hallmarked by elevated cortisol levels, reflecting the severity of illness. Paradoxically, previous studies reported suppressed ACTH, implicating another mechanism driving elevated cortisol during critical illness. We hypothesized that cortisol metabolism is reduced in critical illness, in part via elevated bile acids, which may explain the paradoxical ACTH-cortisol dissociation by negative feedback inhibition.

## Methods

In a first clinical study (*n *= 59), we determined the time course of ACTH and cortisol levels during the first week in the ICU. In a second study (*n *= 28), we calculated the plasma half-life of exogenous cortisol in critically ill patients. In a third clinical study (*n *= 51), urinary cortisol metabolites were quantified to estimate the activity of cortisol metabolizing enzymes. In a fourth study (*n *= 64), we quantified the major cortisol metabolizing enzymes in the liver and adipose tissue in relation to circulating cortisol and bile acids. We performed every study in a similar, heterogeneous ICU population, in comparison with a healthy control group matched for age, gender and BMI.

## Results

In the presence of elevated total cortisol, ACTH remained much lower in patients than in healthy controls (*P *< 0.001), confirming the ACTH-cortisol dissociation during critical illness. Cortisol half-life was substantially prolonged in patients compared to controls. Based on urinary metabolites, the activity of 5α-reductase and 5β-reductase was significantly lower in patients than controls (*P *< 0.0001). Furthermore, the calculated activity of 11-hydroxysteroid dehydrogenase type 2 was reduced (*P *< 0.0001). In the liver, gene and protein expression of 5α-reductase and 5β-reductase was reduced (*P *< 0.0001) and correlated inversely with circulating cortisol. Moreover, the enzyme expression correlated inversely with circulating levels of conjugated bile acids, which were markedly elevated in patients [1] and which have been shown capable of suppressing expression and activity of cortisol metabolizing enzymes [2].

## Conclusion

Reduced expression and activity of cortisol metabolizing enzymes, possibly driven by elevated bile acids, contributes to the hypercortisolism in the critically ill, which explains the increased cortisol plasma half-life and feedback-inhibited ACTH release. Reduced cortisol metabolism could inferentially suppress the cortisol response to an ACTH stimulation test, thereby reducing its diagnostic value for adrenal failure.
